# Ultrasonography for noninvasive and real-time evaluation of peri-implant soft and hard tissue: a case series

**DOI:** 10.1186/s40729-021-00375-2

**Published:** 2021-09-14

**Authors:** Miriam Thöne-Mühling, Oliver D. Kripfgans, Reiner Mengel

**Affiliations:** 1 Department of Prosthetic Dentistry, School of Dental Medicine, Philipps-University Marburg, Georg-Voigt Str. 3, 35039 Marburg/Lahn, Germany; 2 Department of Radiology, BME, and Appl. Phys., University of Michigan, 3218D Med Sci I, 1301 Catherine Street, Ann Arbor, MI 48109-5667 USA

**Keywords:** Ultrasonography, Peri-implant diagnostics, Peri-implantitis, Bone loss, Soft tissue diagnostics

## Abstract

**Background:**

The diagnosis of soft and hard tissue at dental implants will be challenging in the future, as high prevalence of mucositis and peri-implantitis were described in the population. Ultrasonography is a promising non-invasive, inexpensive, painless, and radiation-free method for imaging hard and soft tissue at implants, especially an ultrasound device with a 25-MHz probe demonstrating a high correlation between ultrasound, clinical, and radiological measurements.

**Case presentation:**

The following case series demonstrates the use of ultrasonography with high spatial resolution probe in patients with dental implants affected by soft tissue recession and/or crestal bone loss.

**Conclusion:**

These ultrasound images can provide valuable additional information for the assessment of peri-implant soft and hard tissue.

## Background

The diagnosis of soft and hard tissue at dental implants will be challenging in the future, as high prevalence of mucositis and peri-implantitis were described in the population [18, 45]. More than 40 % of all implants exhibit peri-implant diseases, and almost one in five implants develop increased bone loss at least once after a loading period of 3.4 to 11 years [17]. Patients with periodontal disease in particular experience increased inflammation and bone loss at implants [12, 19].

The two-dimensional intraoral radiograph is the current gold standard in the measurement of the peri-implant bone loss [46, 51]. However, radiographs only show the mesial and distal bone level; the buccal and oral bone is not visible due to the overlay of the implant. Since buccal bone at implants has shown increased bone loss in long-term clinical studies, cone-beam computed tomography (CBCT) and computed tomography (CT) are increasingly used as three-dimensional radiographic imaging procedures [13, 14, 23, 32, 33, 49]. Despite the advantages of three-dimensional imaging, it remains to be noted that bony dehiscence and fenestration on implants cannot be adequately assessed [22, 54]. This is due to the low resolution limit of the image, which does not reliably represent buccal bone in the anterior maxilla, which is usually very thin and less than 1 mm thick [6, 21, 52]. Another disadvantage of three-dimensional images is the susceptibility to artifacts at the transition from metal to bone and soft tissue. This leads to erasure artifacts in imaging, particularly in the diagnostics of titanium implants and their metallic abutments [20, 43, 47]. In terms of radiological exposure, CBCTs generally show reduced radiation exposure compared to CTs [28–30, 42]; however, their radiation exposure is 3 to 40 times higher than that of a two-dimensional panoramic image [30]. Due to the higher radiation exposure, the use of a three-dimensional radiological image is therefore only indicated in selected indications and is not justifiable for periodic, postoperative implant monitoring.

**Fig. 1:**
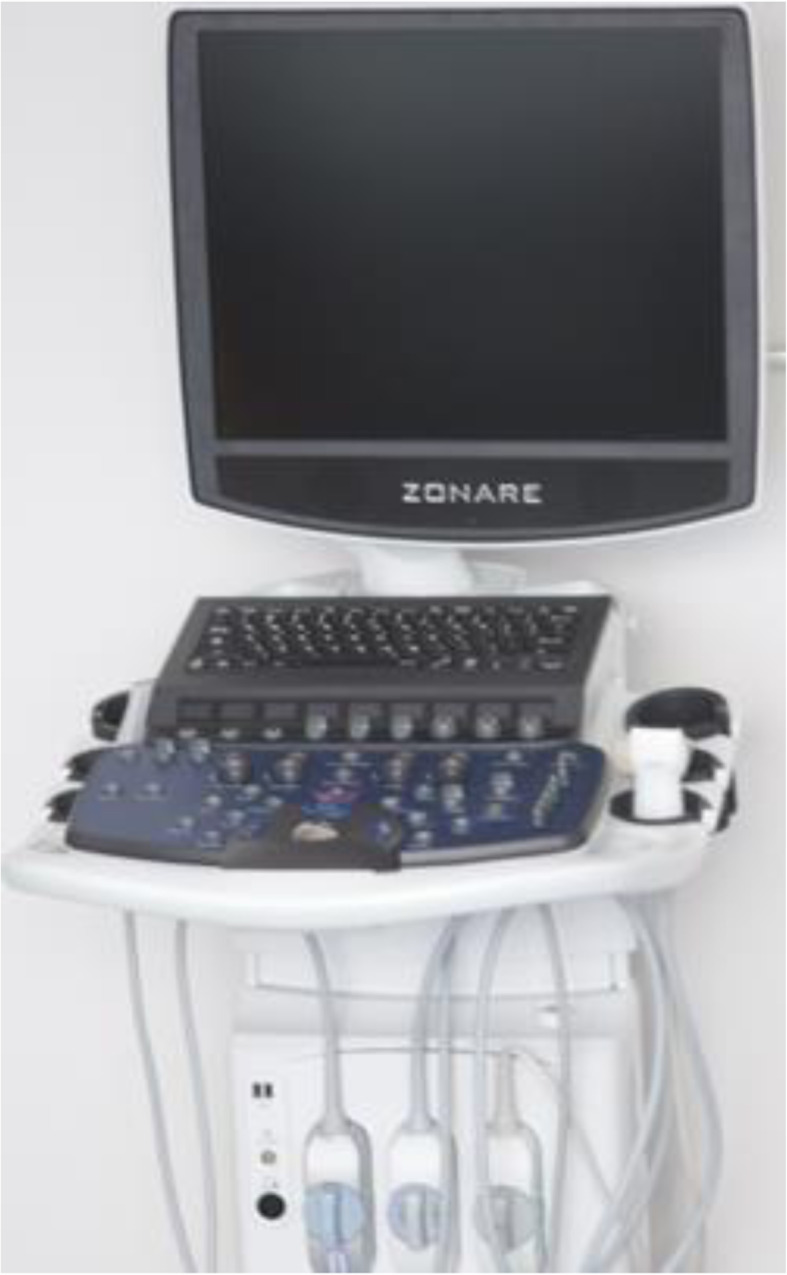
Ultrasound device, model ZS 3 (Mindray Innovation Center, Mindray North America, San Jose, CA, USA)

**Fig. 2:**
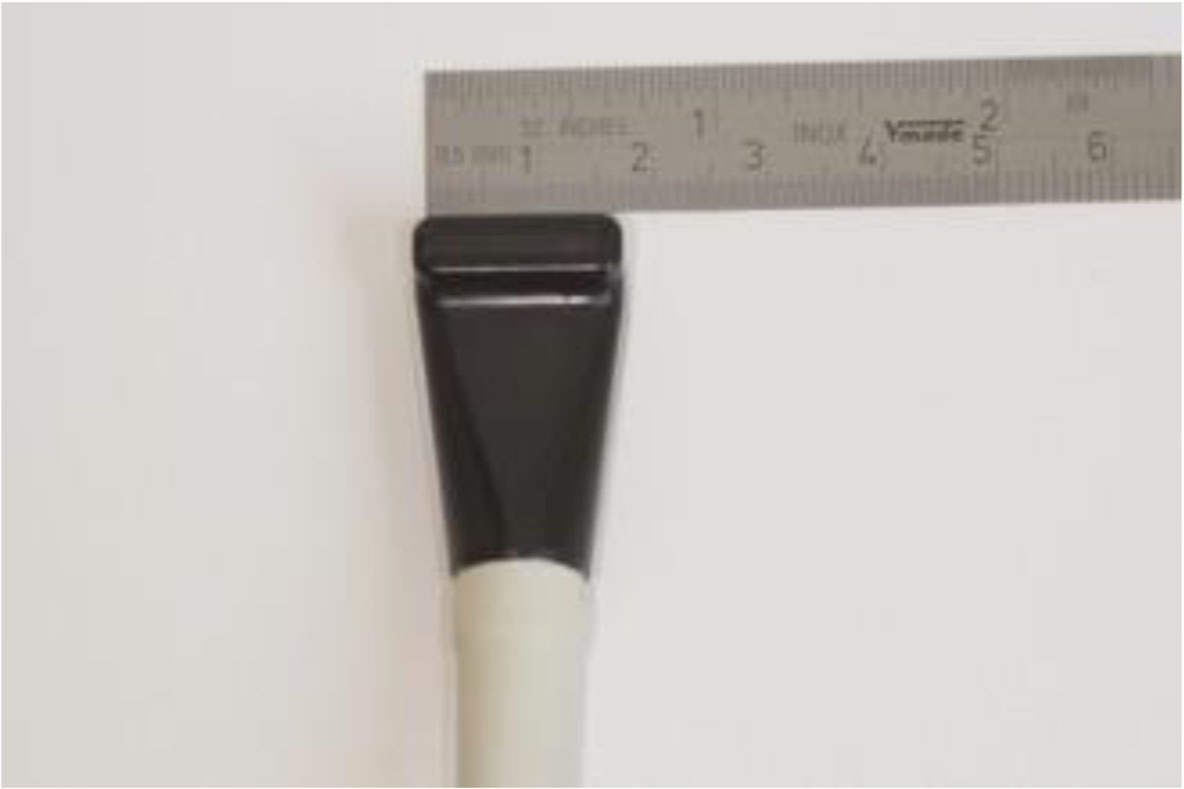
High-frequency probe (up to 60 μm, model L30-8, Mindray Innovation Center, Mindray North America, San Jose, CA, USA) with the size of a toothbrush (30 × 18 × 12 mm)

In addition to radiographs, ultrasonography is a promising non-invasive, inexpensive, painless, and radiation-free method for imaging hard and soft tissue at implants. Especially, an ultrasound device with a high spatial resolution probe (ZS3 system and L30-8 probe, Mindray, Mountain View, CA) the size of a toothbrush (30× 18× 12 mm), co-developed between the University of Michigan and Mindray of North America, demonstrated a high correlation between the ultrasound, clinical and radiological measurements (Figs. 1 and 2). These images can provide valuable additional information for the assessment of peri-implant soft and hard tissue [8–10].

The presented case series of patients with peri-implant soft and hard tissue defects is the first publication of such cases obtained and imaged outside the University of Michigan using ultrasonography with high spatial resolution of up to 60 μm.

## Case presentation

### Case 1

In 2013, a 61-year-old patient (male) was referred to the Dental School of Medicine, Marburg/Lahn, Germany. The general medical history showed no evidence of systemic disease, and according to the self-report, there was no nicotine or alcohol consumption. A familial increased incidence of periodontal disease could not be ruled out because both parents had been edentulous at the age of 40. In the mandible, all teeth except teeth 44 and 45 were not worthy of preservation and removed. After a healing period of 6 months, 3 implants (Nobel Biocare; Nobel Replace Straight Groovy, Zürich, Swiss) in the third quadrant and one implant in the four quadrant with lengths of 10 to 11.5 mm and a diameter of 3.5 or 4 mm were inserted into the mandible according to the traditional delayed protocol. The 2nd stage surgery was performed after a healing period of 3 months.

The prosthetic appliance according to the double-crown concept was provided at the Dental School of Medicine, Philipps-University, Marburg/Lahn [34].

**Fig. 3:**
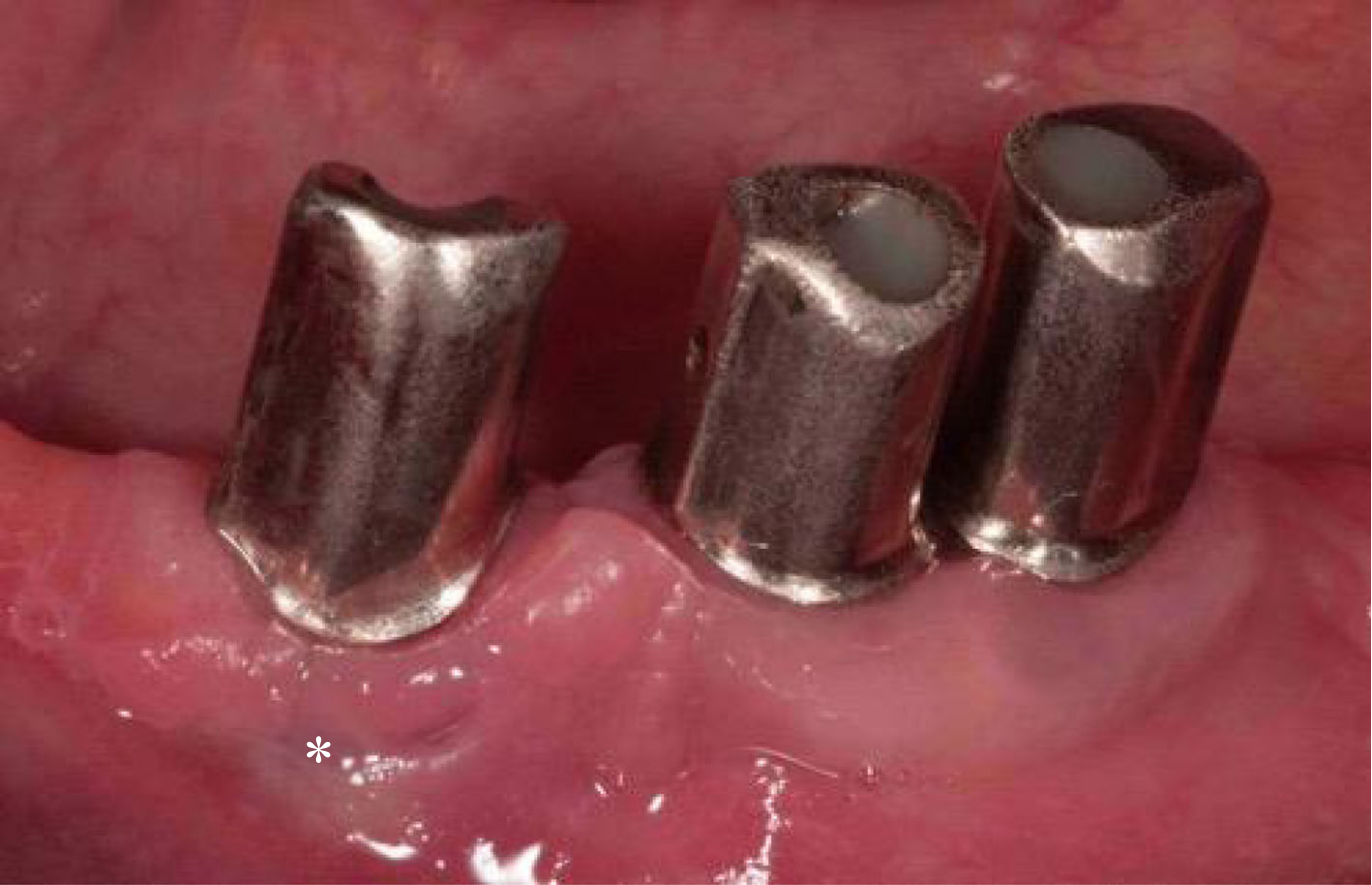
The clinical findings 7 years after insertion of the superstructure showed at the implants with primary crowns no soft tissue recessions and the keratinized mucosa was > 1 mm. The implant in region 33 showed a blue-livid discoloration buccally (marked by an asterisk)

The clinical findings 7 years after insertion of the superstructure showed no soft tissue recessions at the implants with primary crowns and the keratinized mucosa was > 1 mm (Fig. 3). The implant in region 33 showed a blue-livid discoloration buccally.

**Fig. 4:**
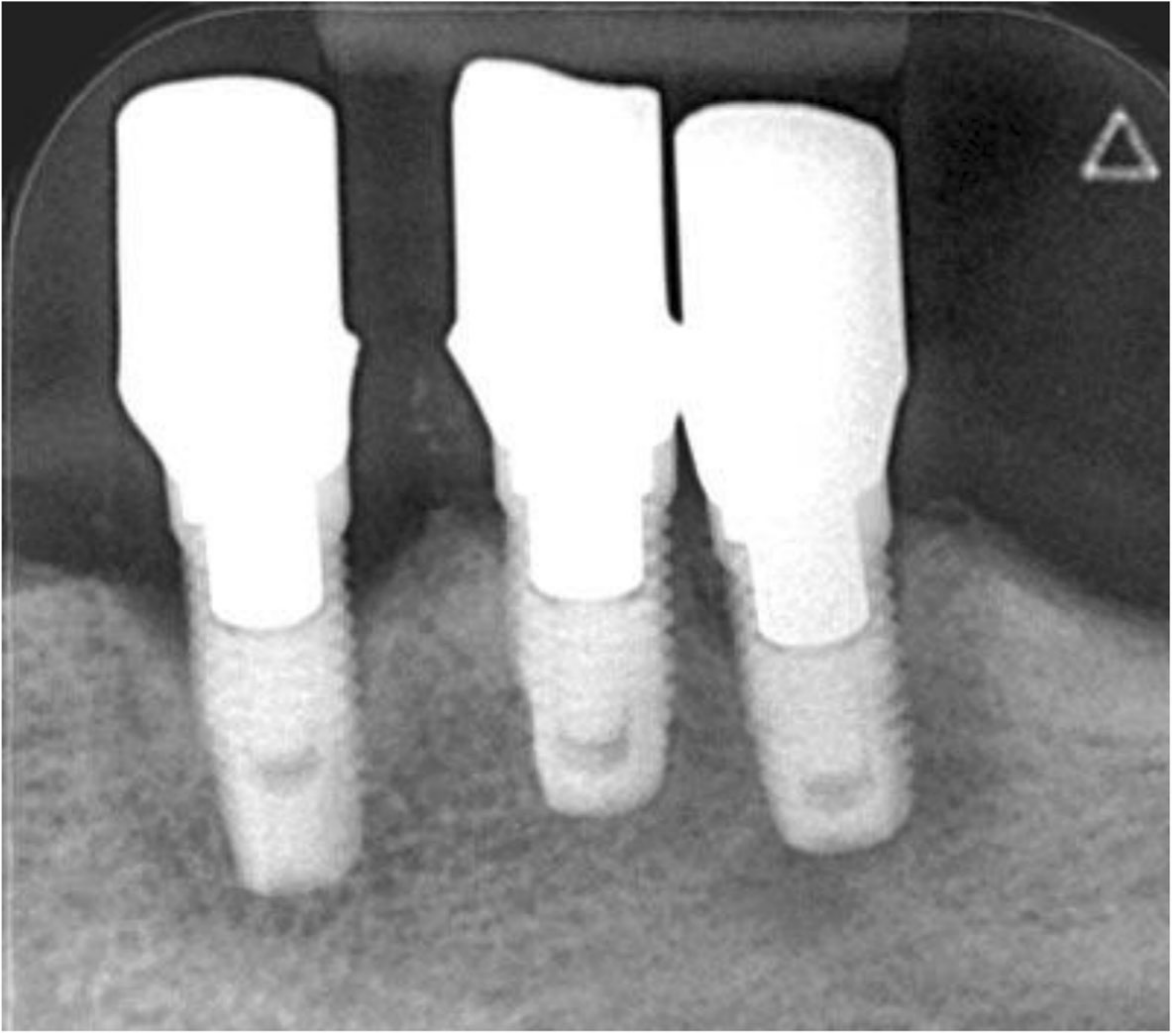
The intraoral radiograph 7 years after insertion of the superstructure showed horizontal and vertical bone loss at all implants. At the implant in region 33, the mesial and distal crestal bone loss reached up to the 6th thread, at implant in region 34 to the 4th thread, and at the implant in region 35 to the 5th thread

The intraoral radiograph using the long-cone technique and the Rinn system (XCP Instruments, Rinn Corporation Elgin, IL, USA) showed horizontal and vertical bone loss at all implants 7 years after insertion of the superstructure (Fig. 4). At the implant in region 33, the mesial and distal crestal bone loss was up to the 6th thread, at implant in region 34 to the 4th thread, and at the implant in region 35 to the 5th thread.

**Fig. 5:**
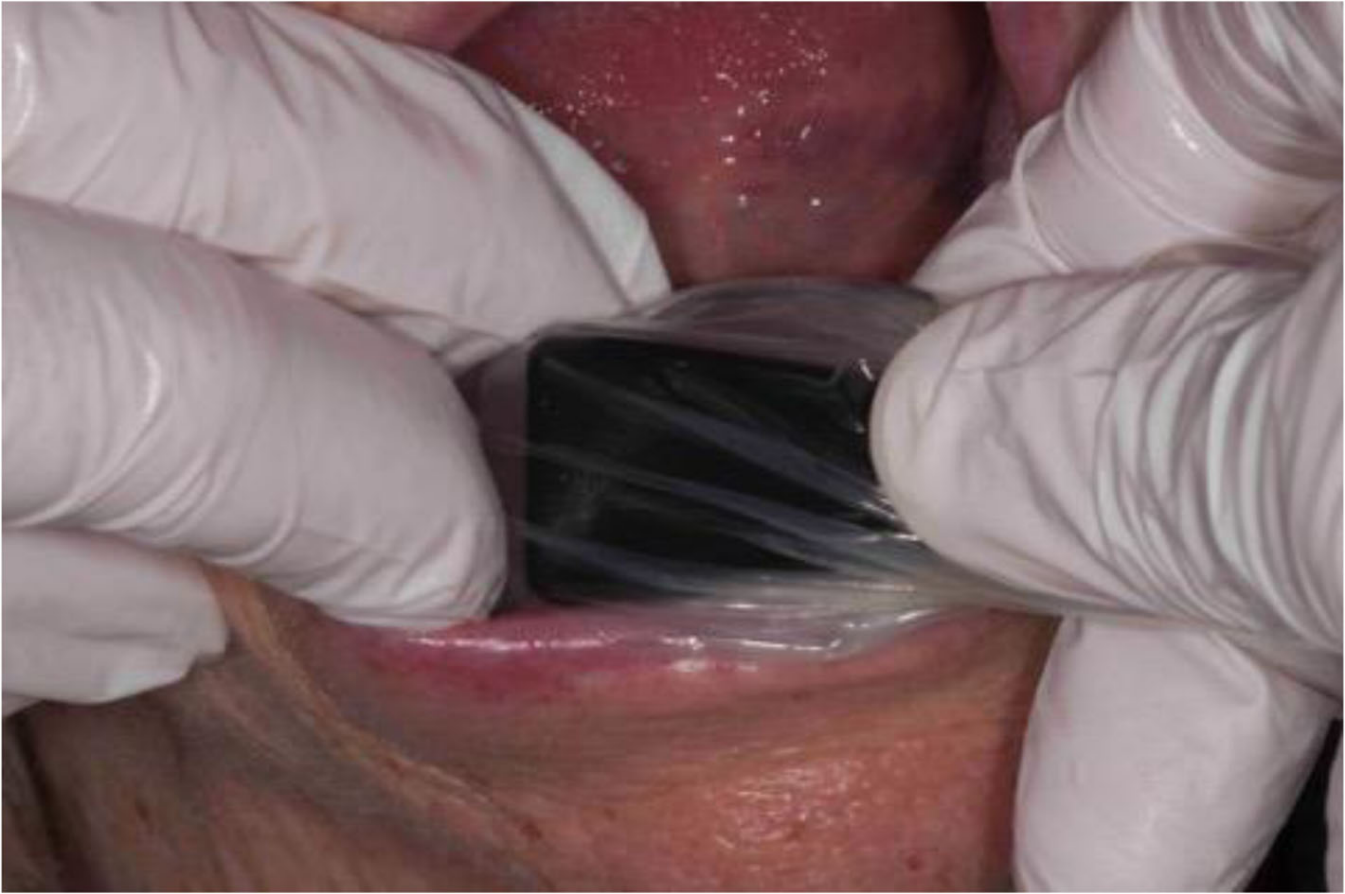
Intraoral placement of the here reported high-resolution ultrasound probe (up to 60 μm) with protective cover and gel standoff pad (not visible)

For ultrasonography the probe was placed buccally as parallel as possible to the longitudinal axis of the implant (Fig. 5). After identification of the implant surface, the probe was cranio-caudally aligned along this longitudinal axis so that parts of the superstructure, the exposed implant surface, and adjacent hard and soft tissue were imaged simultaneously. The intraoral application of ultrasound on implants means imaging of tissue immediately adjacent to the probe aperture. Therefore, a standoff pad (Parker Aquaflex® Ultrasound Gel Pad, New Jersey, USA) was used as forward section, to place the region on interest into the elevational focus of the probe (7 mm). Furthermore, the probe was placed inside a single-use latex cover, which acted as a mechanical barrier for infection control purposes. The thickness of the pad is individually adjustable and the pad is relatively volume stable compared to conventional ultrasound gels. Since the gel pad is homogeneous and free of air inclusions, artifact formation is reduced. In addition, it compensates for unevenness and thus ensures an even pressure distribution.

**Fig. 6:**
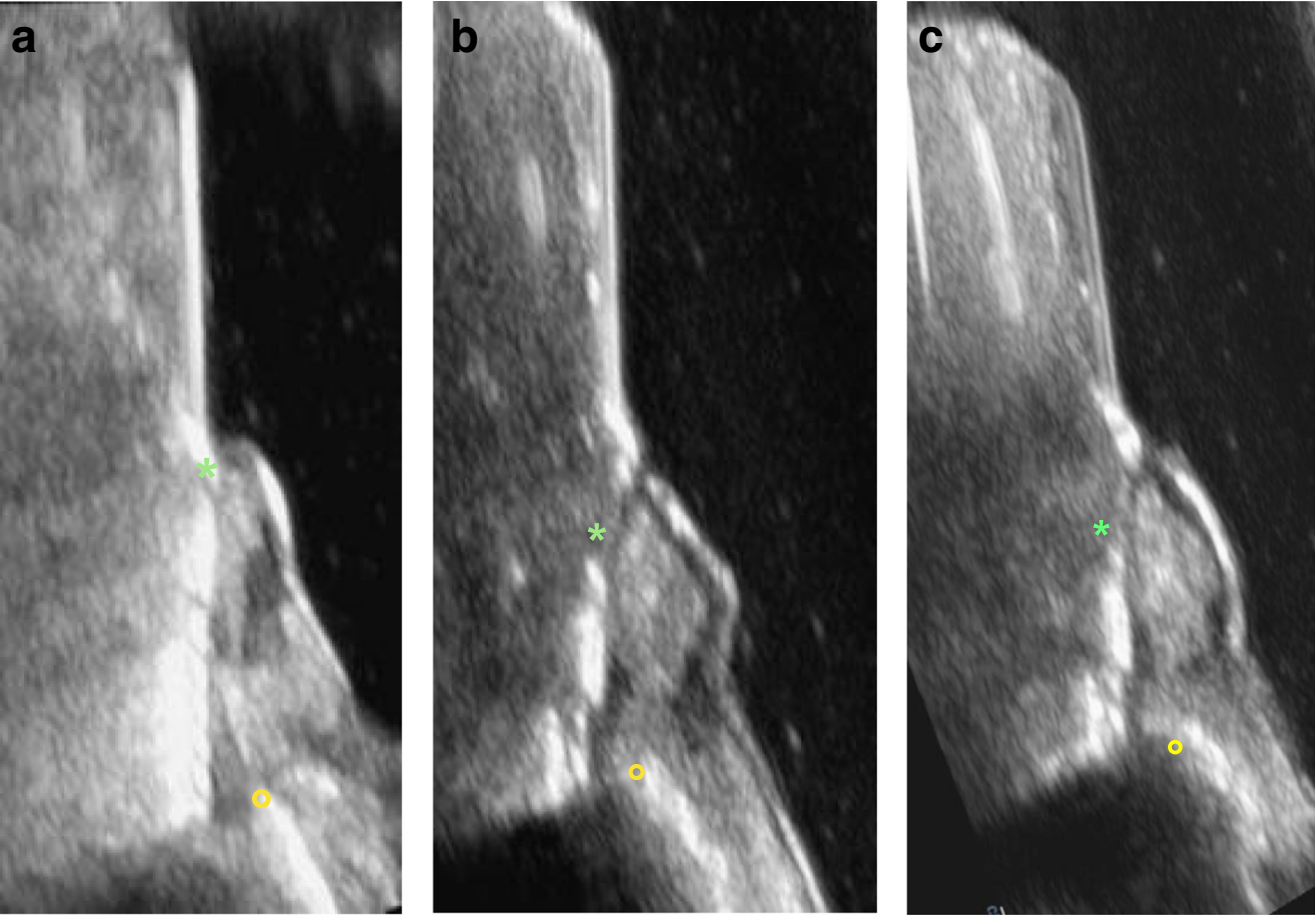
**a** Ultrasonic imaging at the implant in region 33 revealed buccal bone loss up to the 6th thread (asterisk marks the implant-abutment interface; circle marks the bone). **b** Ultrasonic imaging at the implant region 34 revealed buccal bone loss up to the 3rd implant thread (asterisk marks the implant-abutment interface; circle marks the bone). **c** Ultrasonic imaging at the implant region 35 revealed buccal bone loss up to the 2nd implant thread (asterisk marks the implant-abutment interface; circle marks the bone)

Ultrasonic imaging at the implant in region 33 revealed buccal crestal bone loss up to the 6th thread, at implant in region 34 to the 3rd thread, and at the implant in region 35 to the 2nd thread (Fig. 6a–c).

**Fig. 7:**
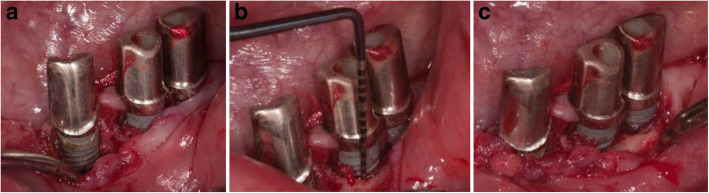
**a** The Intraoperative situation at the implant in region 33 showed calculus and buccal crestal bone loss up to the 6th thread. **b** At the implant in region 34, the buccal crestal bone loss was up to the 3rd thread. The distance from the crestal bone to the abutment implant connection was 6 mm. **c** At the implant in region 35, the buccal crestal bone loss reached up to the 2nd thread

The intraoperative situation at the implant in region 33 showed calculus and buccal bone loss up to the 6th thread (Fig. 7a). At the implant in region 34, the buccal crestal bone loss was up to the 3rd thread. The distance from the crestal bone to the abutment implant connection was 6 mm (Fig. 7b). At the implant in region 35, the crestal buccal bone loss was up to the 2nd thread (Fig. 7c).

### Case 2

In 2004, a 29-year-old patient (male) was referred to the Dental School of Medicine, Marburg/Lahn, Germany. The general medical history showed no evidence of systemic disease, and according to the self-report, there was no nicotine or alcohol consumption. A familial increased incidence of periodontal disease could not be ruled out because both parents had been edentulous at the age of 30. In the maxilla, all teeth were not worthy of preservation and removed. After a healing period of 6 months, 3 implants (Nobel Biocare; Nobel Replace Straight Groovy, Zürich, Swiss) in the first quadrant and 2 implants in the second quadrant with lengths of 10 to 13 mm and a diameter of 3.5 or 4 mm were inserted according to the traditional delayed protocol. The 2nd stage surgery was performed after a healing period of 6 months. The prosthetic appliance according to the double-crown concept and the 3- to 6-month recall schedule was provided at the Dental School of Medicine, Philipps-University, Marburg/Lahn, as described in case report 1.

**Fig. 8:**
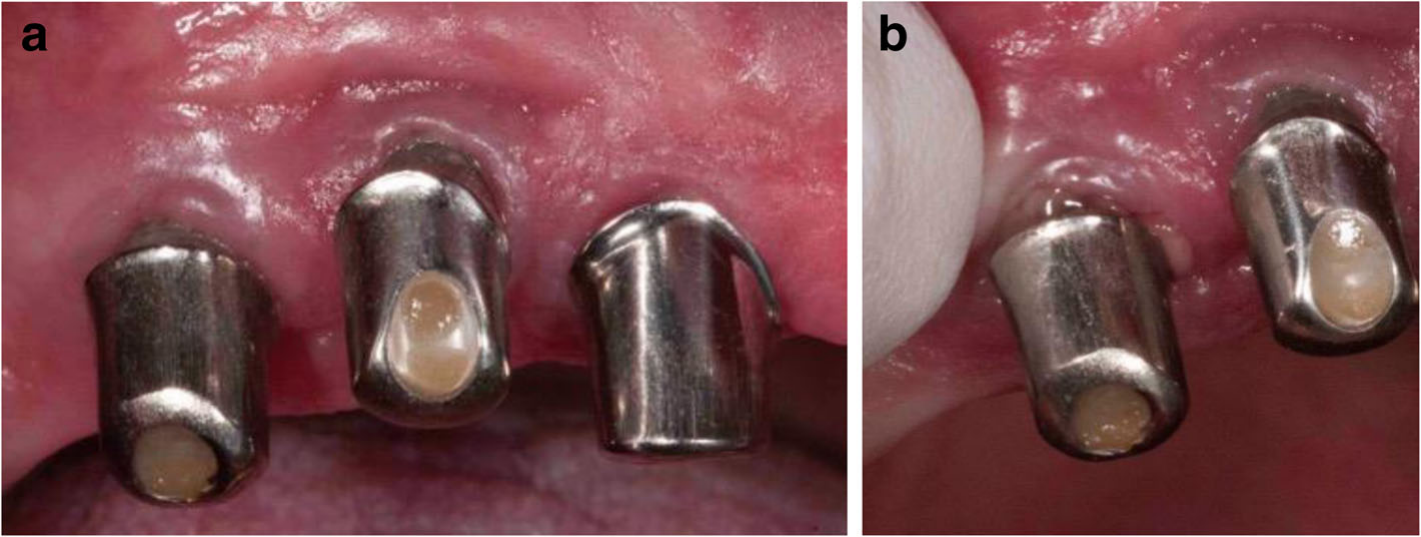
**a** The clinical findings 16 years after insertion of the superstructure showed at the implants with primary crowns slight recessions of the soft tissue and the keratinized mucosa was > 1 mm. **b** The implant in region 14 showed suppuration on light pressure

The clinical findings 16 years after insertion of the superstructure showed at the implants with primary crowns slight recessions of the soft tissue and the keratinized mucosa was > 1 mm (Fig. 8a). The PD at the implants was 6–10mm with BOP and suppuration on light pressure at the implant in region 14 (Fig. 8b).

**Fig. 9:**
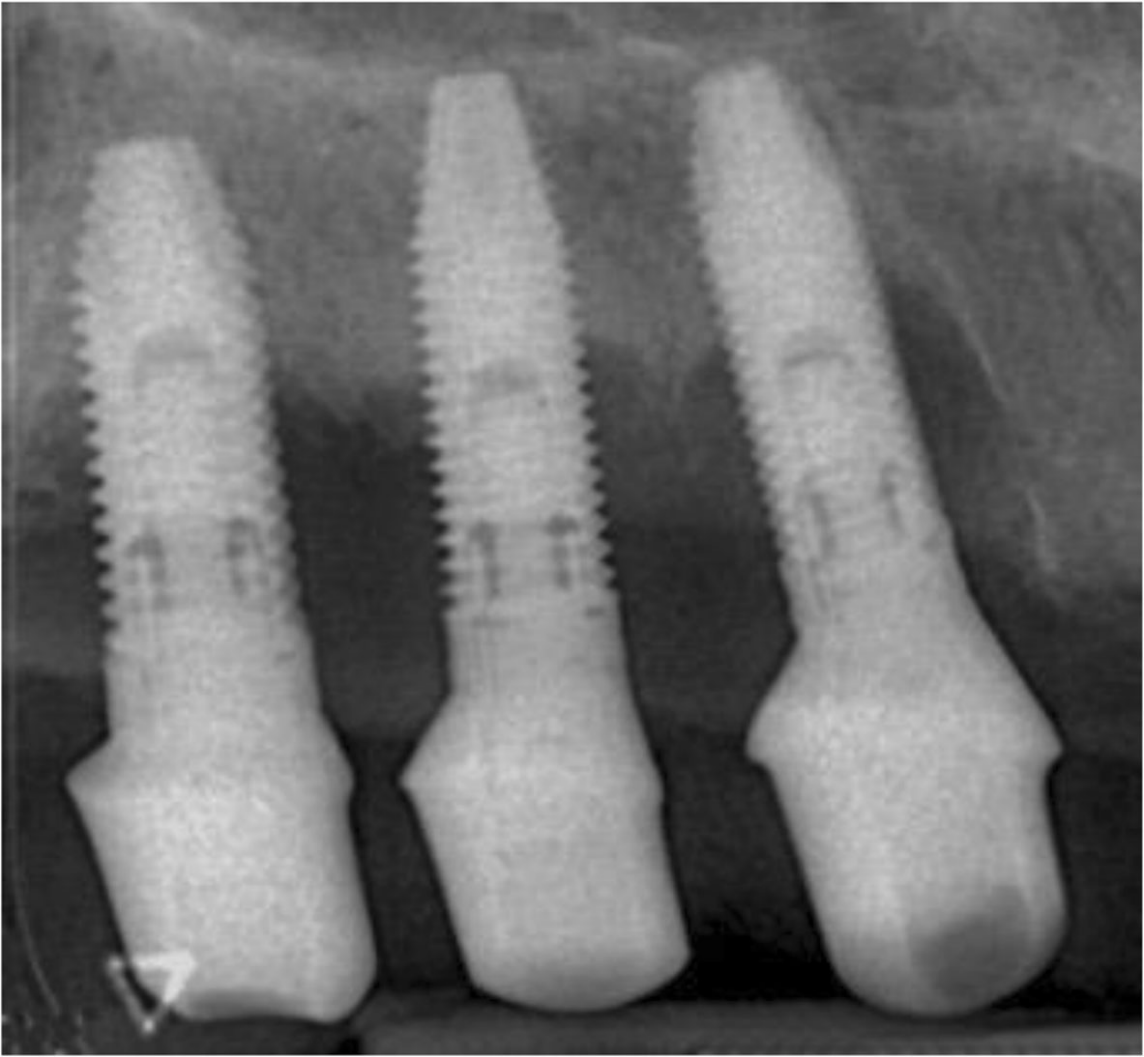
The intraoral radiographs 16 years after insertion of the superstructure showed horizontal and vertical bone loss up to the 10th thread at all implants

The intraoral radiograph 16 years after insertion of the superstructure showed horizontal and vertical bone loss up to the 10th thread at all implants (Fig. 9).

**Fig. 10:**
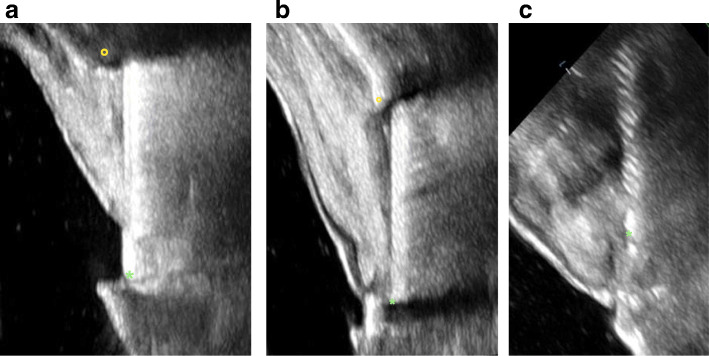
**a** Ultrasonic imaging at the implant in region 14 revealed a buccal bone loss up to the 12th thread (asterisk marks the implant-abutment interface; circle marks the bone). **b** Ultrasonic imaging at the implant in region 13 revealed a buccal bone loss up to the 14th thread (asterisk marks the implant-abutment interface; circle marks the bone). **c** Ultrasonic imaging at the implant in region 12 could not be visualized from the buccal side due to its anatomical situation (subnasal position). Imaging palatal to the implant showed bone loss up to the 13th thread. The palatal image is severely distorted because it was not possible to place the probe parallel to the implant on the oblique palate, and so it was impossible to visualize the apical bone edge (asterisk marks the implant-abutment interface)

Ultrasonic imaging at the implant in region 14 revealed buccal crestal bone loss up to the 12th thread and at implant in region 13 to the 14th thread (Fig. 10a, b). The region of interest at the implant in region 12 could not be visualized from the buccal side due to its anatomical situation (subnasal position). Imaging palatal to the implant showed bone loss up to the 13th thread. The palatal image is severely distorted because it was not possible to place the probe parallel to the implant on the oblique palate, and so it was impossible to visualize the apical bone edge (Fig. 10c).

**Fig. 11:**
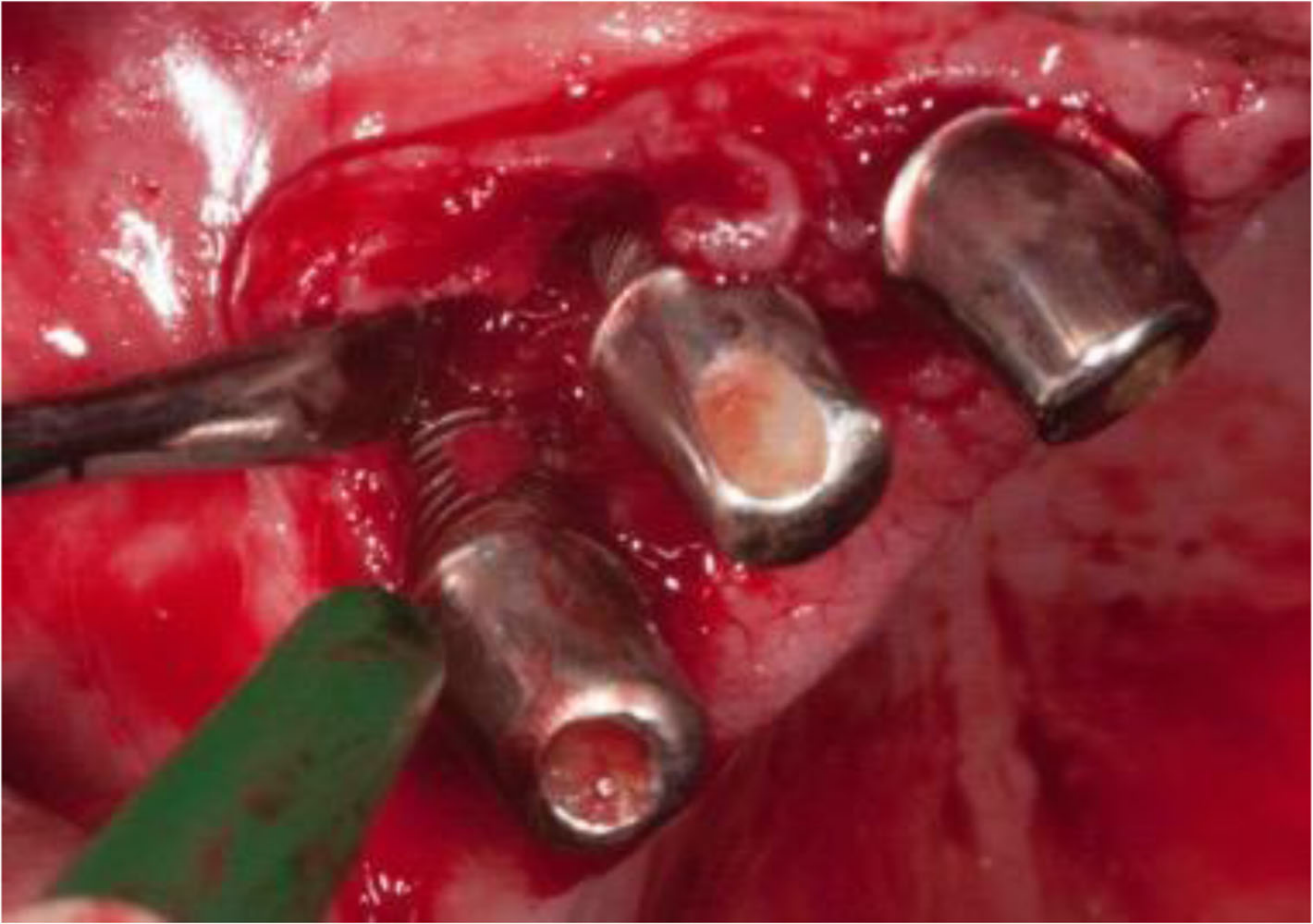
The intraoperative situation at the implant in region 14 and 13 showed buccal bone loss far beyond the 6th thread

The intraoperative situation at the implant in region 14 and 13 showed buccal bone loss far beyond the 6th thread (Fig. 11).

### Case 3

In 2020, a 68-year-old patient (female) was referred to the Dental School of Medicine, Marburg/Lahn, Germany. The general medical history showed no evidence of systemic disease, and according to the self-report, there was no nicotine or alcohol consumption.

In 2014, one implant (Camlog, Conelog, Screw Line, Ø 3.3 mm, length 11 mm) was inserted in the mandible in region 43 according to the traditional delayed protocol. The 2nd stage surgery was performed after a healing time of 3 months. The prosthetic appliance, according to the double-crown concept at the tooth 33 and the implant in region 43, was provided at a private dental office. After insertion of the superstructure, there was no regular recall schedule.

**Fig. 12:**
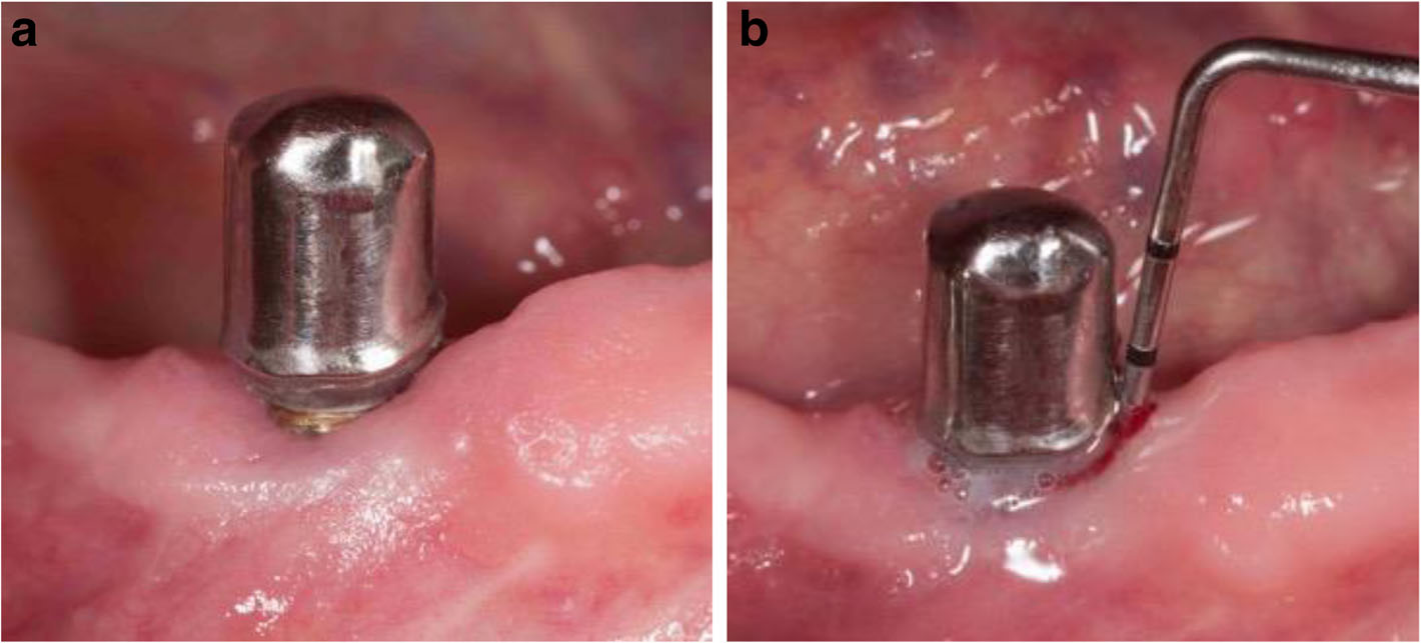
**a** The clinical findings at the implant 6 years after insertion of the superstructure showed a slight buccal recession of the soft tissue. The keratinized mucosa was > 1 mm. **b** The probing depth was 7 mm with BOP and suppuration

Clinical findings at the implant 6 years after insertion of the superstructure showed a slight buccal soft tissue recession; keratinized mucosa was > 1 mm (Fig. 12a). The probing depth was 7 mm with BOP and suppuration (Fig. 12b).

**Fig. 13:**
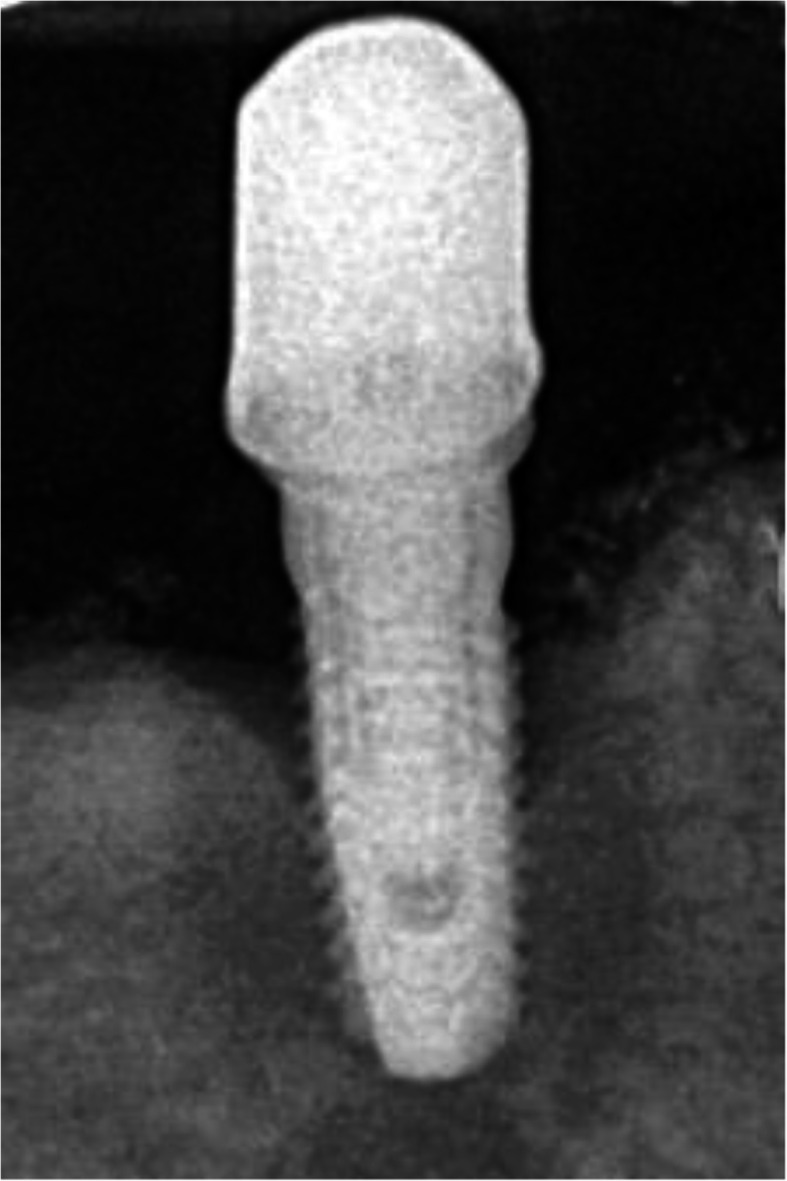
The intraoral radiograph 6 years after insertion of the superstructure showed horizontal and vertical bone loss at the implant. The crestal bone loss was up to the 6th thread.

The intraoral radiograph 6 years after insertion of the superstructure showed horizontal and vertical bone loss at the implant. The crestal bone loss was up to the 6th thread (Fig. 13).

**Fig. 14:**
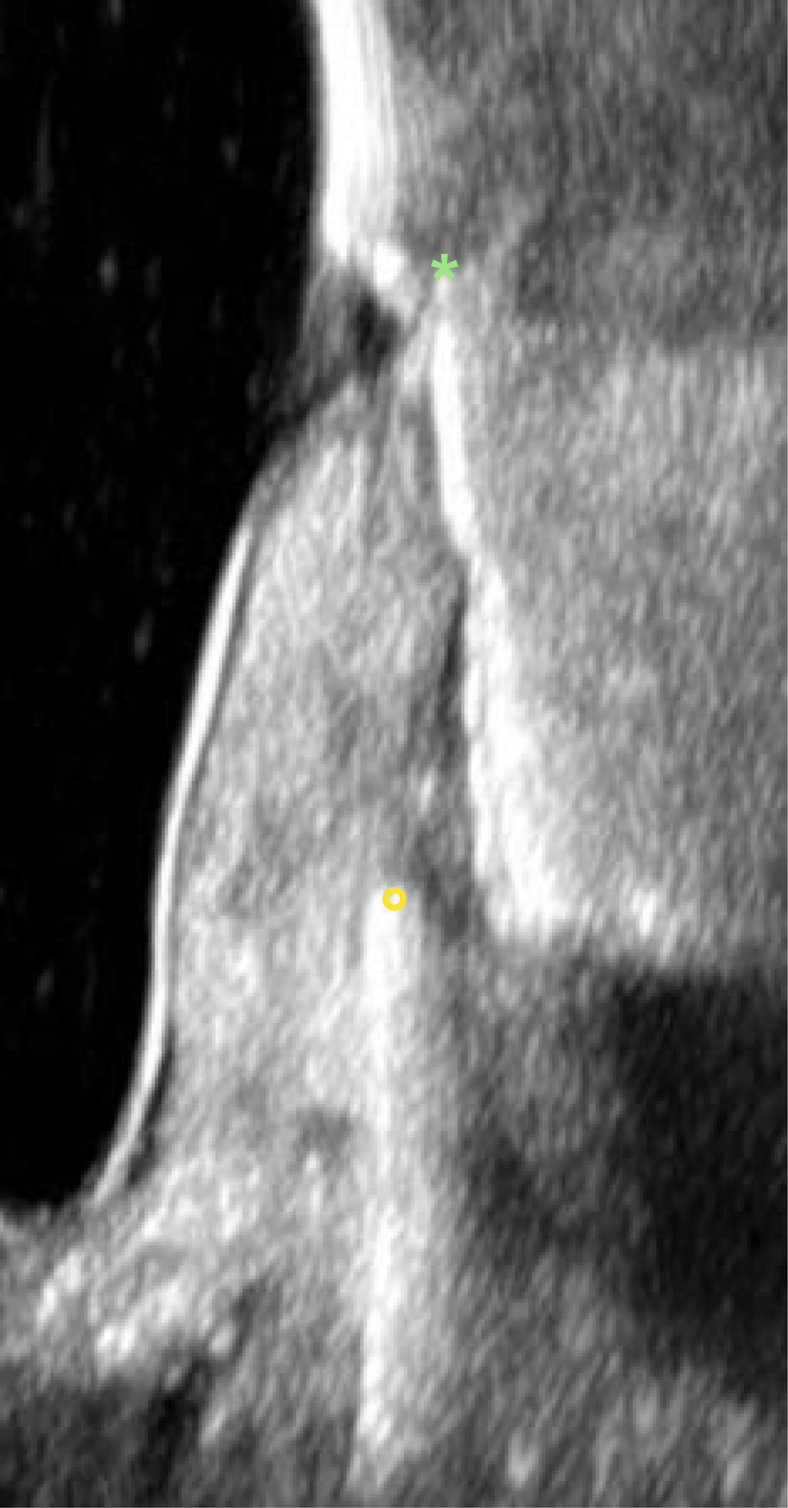
Ultrasonic imaging at the implant in region 43 revealed a buccal bone loss up to the 5th thread with an infracrestal bone loss (asterisk marks the implant-abutment interface; circle marks the bone)

Ultrasonic imaging of the implant in region 43 showed a crestal bone loss buccally up to the 5th thread with a deep infracrestal bone loss (Fig. 14). The implant surface in the infrabony region can system-related not be validly assessed. The free-standing crater-forming bone edge does not seem to be able to completely conceal the underlying defect, but the titanium surface behind is no longer reliably displayed.

**Fig. 15:**
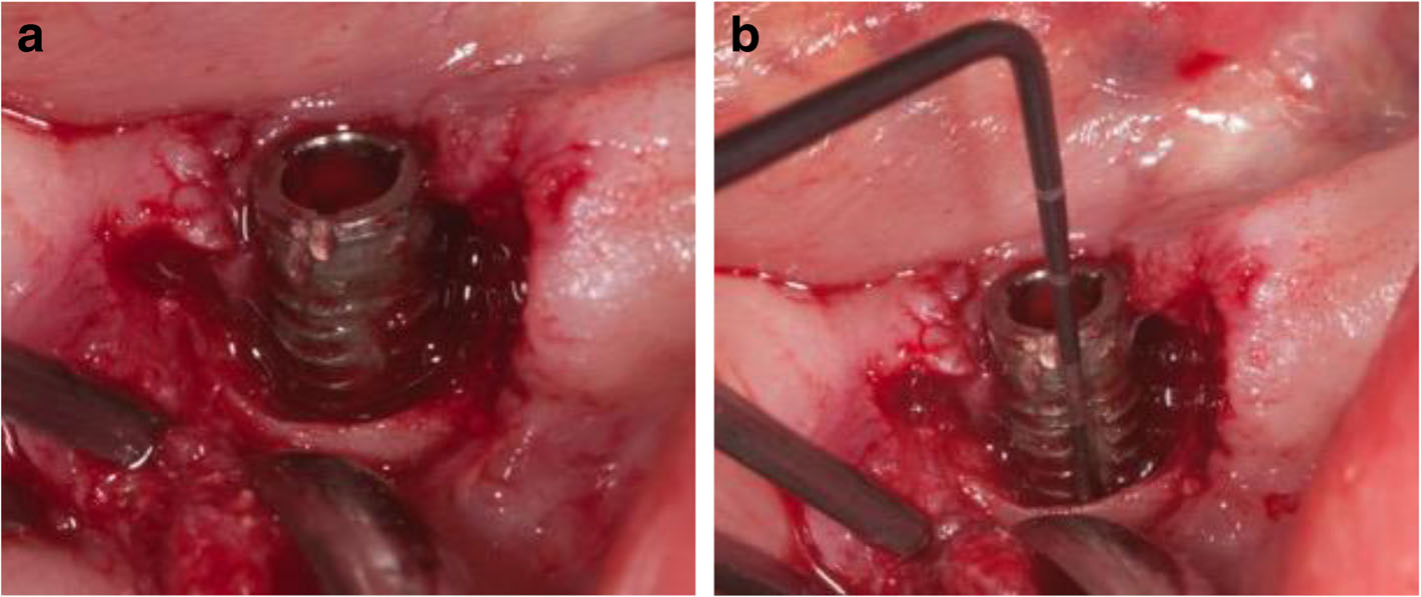
**a** The intraoperative situation at the implant in region 43 reveals a buccal bone loss up to the 6rd thread. **b** The horizontal bone defect is 8 mm, and the vertical defect 3 mm

The intraoperative situation at the implant in region 43 reveals buccal bone loss up to the 6rd thread. The horizontal bone defect is 8 mm, and the vertical defect 3 mm (Fig. 15a, b).

## Discussion

The first data of diagnostic ultrasound in dentistry reported in the literature seems to be in 1963 by Baum et al., who used a 15-MHz transducer with the aim of visualizing the interior structures of teeth [2]. Since then, many different ultrasound applications in dentistry have been reported [31, 39]. For measuring the thickness of periodontal soft tissue, the value of ultrasound images has already been investigated [35–37]. Further studies have evaluated the use of ultrasound in the diagnosis of alveolar bone and tooth structure [3, 5, 16, 27, 38, 40, 41, 44, 50].

The discovery of new applications of ultrasound seems to be ongoing. In 2014, Vollborn et al. [53] developed a high-frequency ultrasonic device for intraoral digitization of prepared teeth to counteract problems due to blood, saliva, and the gingival retraction procedure in case of subgingival preparation margins.

All of these studies demonstrate the promising future of ultrasonography as a diagnostic imaging tool in dentistry. However, many of these studies were performed with large ultrasound probes with a resolution of larger than 75 μm (20 MHz), which are reduced to extra oral use and have limited applicability in routine dental diagnostics [31, 39].

In the presented case series, ultrasonography of soft tissue and crestal bone level was performed with an intraoral capable high-resolution probe (up to 60 μm, frequency range 8 to 30 MHz). In all patients, the soft tissue and bone crest could be localized buccally on almost all implants. These results confirm other clinical studies that have demonstrated accurate soft tissue and bone level imaging using ultrasound scanning with a 25-MHz probe [8, 8]. In one clinical study, the height of the interdental papilla, width and height of soft tissue, width and height of the mucosa above the bone in tooth spaces, and crestal bone level were determined with a 24-MHz ultrasound probe [48]. The aim was to evaluate the correlation and accuracy of ultrasound in measuring periodontal dimensions, compared to direct clinical CBCT methods. The results revealed that ultrasonic imaging can be valuable for accurate and real-time periodontal diagnosis. In another study at implants in human native jaws, the dimension of soft tissue was determined by ultrasonic measurements [8]. The width and height of the soft tissue showed a high correlation with the CBCT and the direct clinical measurement with a periodontal probe.

A systematic review identified several applications of ultrasonography in the implant treatment phases [4]. In the planning phase, vital structures as well as soft and hard tissue biotype can be identified by ultrasound. Furthermore, the ridge width, bone density, and cortical bone thickness may be evaluated. In the surgical phase, vital structures can be identified and primary stability can be indicated. In the follow-up phase, the crestal bone level around implants can be evaluated and implant-bone stability can be indicated.

These results demonstrate that intraoral ultrasonic imaging can be used predictably for soft tissue diagnostics at teeth and implants without concerns about ionizing radiation. In particular, prior to surgical treatment for bone and soft tissue regeneration, ultrasonography of the soft tissue could provide valuable additional information, since the success rates are influenced by the soft tissue biotype [11]. Ultrasonography could also be valuable in peri-implant diagnostics, since sufficient width and thickness of soft tissue is a prerequisite for the long-term success of implants and can be surgically optimized in case of a diagnosed deficiency [15, 24–26]. Furthermore, in the three-dimensional planning of implants, the imaging of the crestal bone with overlying soft tissue can improve the predictability of an esthetic and functional emergence profile of prosthetic restorations.

In addition to the imaging of crestal bone level and soft tissue dimensions, the validity of ultrasonography to determining the lingual nerve in human cadavers and live humans was tested [1]. The results showed that ultrasound accurately measured mandibular lingual soft tissue structures on cadavers and the lingual nerve on live humans.

In addition to morphological and topographical imaging of the soft tissue, a recently published study investigated functional ultrasonography with the same probe as here reported [8]. In one patient, each with a healthy implant, an implant with mucositis and peri-implantitis, and an implant with soft tissue and bone loss without signs of inflammation, blood flow to the peri-implant soft tissue was visualized. The results showed that the ultrasonic imaging not only provides anatomical cross-sectional images, but also can show the blood flow in the soft tissue. This functional imaging mode (color flow and color power mode), in conjunction with photoacoustics, could be of particular interest in the future in order to differentiate in peri-implant tissue the changes in active blood vessels with respect to the ratio of oxygenated/deoxygenated hemoglobin and total blood volume. It should be further investigated whether such image modality combination could be used to differentiate disease activity such as different degrees of severity of peri-implantitis.

Despite the promising results with the reported high-frequency probe, ultrasonography has shown limitations in the diagnostic value of soft tissue and crestal bone level. For example, the extent and severity of crestal bone loss may influence the validity of ultrasonic imaging, since advanced bone loss (> 6 mm) is expected to be less accurate than moderate bone loss [8]. Furthermore, infrabony defects at implants or teeth cannot be determined predictably. Application-related limitations in ultrasonography include the dependence of image quality on the technical skills of the practitioner. Certainly, clinicians will need training, either by means of continuing education or via specialty training. First of all, a coupling medium between the probe head and the surface to be scanned is required to reduce the display of air bubbles in the ultrasound images. The probe should be placed as parallel as possible to the longitudinal axis of the implant in order to prevent distortions and to get a true-scale image. In some cases, anatomical obstacles impede an appropriate probe alignment.

## Conclusions

These case series demonstrate that an ultrasound device with an intra-orally functional, high-resolution probe can be used predictably for imaging of the crestal bone level and soft tissue dimensions at dental implants. Further clinical studies should evaluate the applications of non-invasive, high-resolution ultrasonography in the routine diagnostics in dentistry.

## Data Availability

Not applicable.
